# Identification of Subvisible Particles in Biopharmaceutical Formulations Using Raman Spectroscopy Provides Insight into Polysorbate 20 Degradation Pathway

**DOI:** 10.1007/s11095-015-1670-x

**Published:** 2015-03-14

**Authors:** Miguel Saggu, Jun Liu, Ankit Patel

**Affiliations:** Late Stage Pharmaceutical Development, Genentech Inc., South San Francisco, California 94080 USA

**Keywords:** degradation, fatty acid, particle identification, polysorbate 20, Raman spectroscopy

## Abstract

**Purpose:**

To study composition and heterogeneity of insoluble subvisible particles in Mab formulations resulting from degradation of polysorbate 20 and to develop a better understanding of the mechanisms of polysorbate degradation leading to particle formation.

**Methods:**

In this study, we exploit the potential of Raman microscopy for chemical identification of particles in monoclonal antibody formulations. Through a combination of experiments and density functional theory (DFT) calculations, we identified unique spectral marker bands for insoluble degradation products of polysorbate 20. We first applied our methodology to identify particles in model systems containing complex mixtures of fatty acids and then to subvisible particles in antibody formulations stored at 5°C for several years.

**Results:**

Most of the subvisible particles identified were comprised of mixtures of fatty acids with no observable signal from fatty acid esters consistent with hydrolysis being the predominant degradation mechanism leading to particulate formation under these storage conditions.

**Conclusions:**

Our methodology is generally applicable for identification of particles in antibody formulations and, in particular, has the potential to give detailed information about particle heterogeneity and insight into mechanistic aspects of particle formation.

**Electronic supplementary material:**

The online version of this article (doi:10.1007/s11095-015-1670-x) contains supplementary material, which is available to authorized users.

## Introduction

Control of particles in biopharmaceutical formulations is of particular importance when developing the manufacturing process, assessing product quality, and addressing safety concerns (1). There has been a significant amount of effort spent on the quantification of subvisible particles in protein therapeutics as regulatory health authorities expect biopharmaceutical companies to characterize the occurrence of particles >10 μm and >25 μm as outlined in the pharmacopeias, *e.g.,* USP <787 > and USP <788 > (2,3). Recently, concerns have been raised that proteinaceous subvisible particles may trigger immunogenic responses, but the roles of particle chemical composition and structure in generating an immune response are under debate as these attributes are particularly difficult to characterize (2,4). It is noted that all biotherapeutics contain subvisible particles, most of which are not harmful and well within the USP specification (4). For products containing high or varied particle counts, identification of particles is key in assessing potential mechanism and impact on product quality.

Subvisible particles in protein formulations mostly show a continuous size distribution that can range from a few microns to hundreds of microns (4,5). Particles with a size smaller than one micron are considered submicron particles and are especially difficult to count and characterize. There are only few techniques commercially available to study submicron particles such as nanoparticle tracking (NanoSight®) or microchannel resonator (Archimedes®), but these possess limited accessible particle size and concentration ranges as well as other technical limitations (6,7). Promising results in distinguishing proteinaceous particles from silicone oil have been obtained using the microchannel resonator, but in general, routine characterization of submicron particles is not yet possible (6). Characterization of subvisible particles is mostly performed using optical techniques, which rely on good optical contrast between the particles and the solution. Over the last few years, flow microscopy techniques such as Micro-Flow Imaging® or FlowCAM® were introduced and are being evaluated to count subvisible particles >1 μm and provide morphology data of particles >5 μm (note that the lower size limit depends on the optics and flow cells utilized in addition to the optical contrast between the particles and the solution) (8). Particle identification based on morphology using flow microscopy allows for discrimination of air bubbles and silicone oil from proteinaceous and foreign particles (8,9). Flow imaging techniques, however, lack the ability to provide information about the exact chemical identity of the investigated particles and their heterogeneity. Techniques that give information about the chemical composition of subvisible particles are limited to electron microscopy (SEM-EDX for inorganic compounds) and vibrational spectroscopy (10). Two types of vibrational spectroscopy are frequently employed for particle identification: Fourier transform infrared spectroscopy (FTIR) and dispersive Raman spectroscopy (10,11). For routine analysis, FTIR spectroscopy is usually employed because of its versatility and easier handling. The drawback of FTIR spectroscopy is its inherent sensitivity to water both in the atmosphere as well as in aqueous solution resulting in interference and low-quality data. This is particular true for smaller size particles where signal-to-noise is very low. The lower limit of detectable particle size using IR reflection is in the 10–20 μm range and not sufficient to cover the typical particle size range for our products (4). On the other hand, dispersive Raman spectroscopy is advantageous for studying biological systems because water shows only minor Raman activity and the use of lasers enables detection of smaller size particles (possibly as low as 0.5 μm for strong scattering molecules such as metal complexes) (10). However, sample acquisition is more demanding and care must be taken to avoid laser-induced photo-damage of the sample. In addition, multi-laser configurations are necessary to optimize Raman scattering and minimize background fluorescence.

The major types of particles occurring in pharmaceutical formulations are usually classified as extrinsic, intrinsic and inherent particles (4). Intrinsic particles are those that are unintentionally introduced during the manufacturing process or during non-sterile sample handling. This category usually includes glass, metal pieces and fibers such as cellulose. The difference between intrinsic and extrinsic particles is that the latter ones are not process-related. Inherent particles are product-related and comprised of degradation products of excipients and proteins in formulations.

Most work focuses on characterization of extrinsic particles. In recent years, product-related particles have gained wide attention due to the potential concerns about immunogenicity of protein particles (2). Within this frame, recent reviews have highlighted the potential impact of excipient degradation, *e.g.,* polysorbate 20 degradation, on protein stability (12,13). Polysorbate 20 is a commonly used surfactant in protein formulations and is typically used to stabilize protein pharmaceuticals against various stresses such as agitation-induced aggregation or surface adsorption (13). It is a chemically diverse mixture containing mainly sorbitan polyoxyethylene fatty acid esters. The composition of the fatty acid side chains varies from lot to lot with lauric acid (40–60%), myristic acid (14–25%), palmitic acid (7–15%) and capric acid (≤10%) being the main species. It is known that polysorbates can degrade *via* two known pathways: hydrolysis and autooxidation (12,13). Hydrolysis creates fatty acids, which are prone to form insoluble particles (12,13). Autooxidation is based on a radical mechanism and hence leads to a variety of degradation products, *e.g.,* peroxides or aldehydes, all of which might affect protein stability. In addition, some of the fatty acid esters, which also result from autooxidation, are insoluble (13). A decrease in polysorbate 20 levels during storage is a potential concern for protein stability due to decreased protection against interfacial stress and potential interaction of degradation products with active antibody, which may lead to proteinaceous particles. Autooxidation is significantly accelerated when formulations are stored at high “stress” temperatures while hydrolysis has been implicated in mildly acidic conditions even at relatively low temperatures (13). However, the factors that influence each degradation pathway in protein formulations are still under debate and there is yet no routine method to identify these types of particles (12,13). Further, characterization of fatty acids and its derivatives is particularly challenging due to the limited stability of these particles in complex mixtures. They have low melting points and are readily resolubilized at ambient temperature. Hence, sample preparation and investigation require stabilizing conditions such as low temperature. Previous studies employed FTIR spectroscopy for particle identification but spectra in the fingerprint region are usually cluttered and do not allow for individual identification of highly similar compounds, in particular in complex mixtures (14).

In the current study, we exploit the potential of dispersive Raman spectroscopy at cryogenic temperatures for the identification of subvisible labile particles in biopharmaceutical formulations resulting from degradation of polysorbate 20. Particles resulting from degraded polysorbate 20 after long-term storage of antibody formulations beyond shelf-life were composed of mixtures of fatty acids consistent with a hydrolytic mechanism leading to particle formation. We developed a methodology, which is generally applicable to study insoluble degradation products and may be applied to other types of particulates.

## Experimental

### Materials

Purified dry capric acid, lauric acid, myristic acid, palmitic acid and ethylene glycol monolaurate with purity >98% were purchased from Supelco (Bellefonte, USA). Polysorbate 20 was purchased from Croda International PLC (Snaith, UK). Hydrated fatty acid particles were prepared by stirring dry fatty acids as received in water for 30 min. The particles were filtered through a gold-coated polycarbonate filter (filtr.AID with 5 μm pore size from rap.ID, Germany) and washed with 2 mL water.

Enzymes for digestion were obtained from Sigma-Aldrich (St. Louis, Missouri). Hydrolysis was performed by digesting 0.2 mg/mL polysorbate 20 in 20 mM sodium acetate at pH 5.5 with 30 ppm rabbit liver esterase or 30 ppm pancreatic lipase at 40°C for 12 h. The reaction was stopped by storing samples at 5° where the reaction rate was reduced to essentially zero.

Monoclonal antibody was produced by Genentech Inc. (South San Francisco, California). MAb1 and Mab2 were formulated at 30–50 mg/mL with typical biological buffers containing sucrose and 0.02% polysorbate 20 (*w/v*). Mab1 was stored at 5°C for 4 years while Mab2 was stored at 5°C for 6 years. This is well beyond the typical shelf-life of a product stored at 2–8°C, which is usually 2–3 years. Both samples showed significant polysorbate 20 degradation (see Results and discussion).

### HPLC to Quantify Polysorbate 20 Degradation

High-performance liquid chromatography was performed on an Agilent Infinity 1260 setup equipped with a temperature-controlled autosampler, column oven, binary gradient pump and evaporative light scattering detector (ELSD, 380-LS, Varian). The ELSD was used with a gas flow rate of 1 standard liter per minute (SLM), nebulizer temperature of 45°C and evaporation temperature of 100°C. The column temperature was set to 30°C. A gradient using two mobile phases as described earlier was used (15). In brief, mobile phase A contained 2% formic acid in water and mobile phase B 2% formic acid in isopropanol. Intact polysorbate 20 was quantified by loading 20 μL of sample onto a Waters Oasis MAX cartridge column (20 mm × 2.1 mm, particle size 30 μm). The flow rate was set to 1 mL/min starting with 90% of mobile phase A. After 1 min, mobile phase B was increased to 20%. After 3.4 min, mobile phase B was increased to 80% and after 3.5 min, to 100%, where it was kept for 0.9 min. After 4.7 min, mobile phase B was set to the initial condition of 10%. Intact polysorbate 20 elutes as a single peak at approximately 4.6 min.

### Raman Spectroscopy

Particles from aged antibody samples were isolated by filtering 0.5–1 mL cold sample through a gold-coated polycarbonate filter (filtr.AID with 5 μm pore size from rap.ID, Germany) and washing with >2 mL ice-cold water. Filters were either immediately dried in a desiccator over phosphorous pentoxide for 2 h to remove residual water or directly transferred to a cold cryo-cell without drying (see below). Vibrational spectra were collected on a Morphologi G3-ID dispersive Raman microscope equipped with a 785 nm diode laser (Malvern Instruments Ltd, UK). The setup is comprised of a RamanRxn1 spectrometer (Kaiser Optical Systems, Inc., USA) and a CFI 60 brightfield/darkfield microscope (Nikon Corporation, Japan). The light was focused through a 50X objective to a spot size of 3 μm and the resulting laser power at the sample was either 4 mW in low power mode or 16 mW in high power mode as controlled with a power meter. Scattered light was detected using a high-performance CCD camera. Raman spectra were collected in the spectral region between 150 and 1850 cm^−1^ with a spectral resolution of 4 cm^−1^. Exposure time varied depending on the nature of the particles and was usually between 60 s and 5 min to minimize photodamage.

Low temperature experiments were carried out using a FDCS196 cryostage (Linkam Scientific Instruments Ltd, UK) equipped with a quartz window in which the gold-coated filter containing particles was enclosed and cooled to a temperature between T = −40°C to T = −120°C by means of nitrogen gas.

### Theory

Quantum chemical calculations were performed using Gaussian 09 (16). Geometries of the molecules were optimized in the gas-phase using the B3LYP functional and 6-311++G(d,p) basis sets (17,18). Vibrational frequencies were calculated using the harmonic approximation and scaled using a wavenumber linear scaling procedure according to Yoshida, *et al.*, with

$$ {\tilde{\nu}}_{obs}=\left(1.0087-0.0000163\cdot {\tilde{\nu}}_{calc}\right)\cdot {\tilde{\nu}}_{calc} $$

where ${\tilde{\nu}}_{obs}$ and ${\tilde{\nu}}_{calc}$ are the observed and the calculated Raman shifts, respectively (19).

Gaussian output consists of Raman activities, A_k_, rather than Raman intensities I_k_. To obtain Raman intensities, the Raman activities were converted using a frequency dependent factor

$$ {I}_k=\frac{{\left({\omega}_0-{\omega}_k\right)}^4}{\omega_k\left(1- \exp \left[-\frac{h{\omega}_k}{k_BT}\right]\right)}\cdot {A}_k $$

where h is Planck’s constant, k_B_ the Boltzmann constant, ω_k_ the vibrational frequency of mode k, and ω_0_ the frequency of the incident laser light (785 nm laser exCitation) (20). The temperature, T, was chosen to be 293 K.

## Results and Discussion

Degradation of protein as well as excipients in biopharmaceutical formulations can lead to formation of insoluble species and the observation of particles. Understanding mechanisms of degradation, in particular of excipients used to stabilize antibody against various stress conditions, is crucial for product quality and protein stability. In this study we focus on insoluble degradation products of polysorbate 20, a surfactant commonly added to formulations to protect antibody against interfacial stress conditions. Polysorbate 20 can form different insoluble degradation products such as fatty acids or fatty acid esters, which are indicative for the mechanism of degradation. To distinguish highly similar molecules using vibrational spectroscopy it is important to identify unique spectral marker bands for each compound first using a combination of experiments and theory. Since the microenvironment of each molecule in the particles can be different than the local structure in the reference compound it is crucial to identify any changes in vibrational spectra, *e.g.,* changes in hydrogen bonding.

In this study, we present an analytical approach to elucidate the chemical composition of visible particles, as well as subvisible particles, by direct spectroscopic analysis of particles captured on filters. This approach can be used to elucidate the mechanism of degradation of polysorbate 20 in model systems and two antibody formulations and is generally applicable to similar systems. First, a spectral library of known degradation products was created. Then, unique spectral marker bands for some of the potential degradation products were identified by analysis of pure reference compounds. Control experiments were performed to evaluate changes in vibrational spectra of the dry reference compounds upon transition into the hydrated state in which particles can be typically found. This ensures that bands associated with different hydration states are not mistaken for unique signature bands for a particular degradation product. In, addition, comparison of experimental data with calculated spectra obtained from quantum-chemical calculations using density functional theory (DFT), allowed confirmation of reliable marker bands. The calculations were able to distinguish similar compounds and to rule out the presence of other polysorbate 20 degradation products in the experimental data.

We then collected Raman spectra on particles generated in two model systems in which polysorbate 20 was digested with different enzymes that both hydrolyze the ester bonds between the sorbitan moiety and the fatty acid side chains resulting in insoluble particles of complex composition, *i.e.,* mixtures of fatty acids. Finally, we characterized subvisible particles from two different biopharmaceutical formulations, which were stored at low temperature over an extended period of several years beyond typical shelf-life and had shown a significant loss of intact polysorbate.

### Raman Spectra of Dry Fatty Acids

In order to create a spectral library that includes reference spectra, Raman spectra of purified dry fatty acids were collected. The fatty acid particles were placed on a clean gold-coated polycarbonate filter. Raman scattering scales with the frequency, ν, of the incident laser beam as ~ ν^4^. To achieve maximal scattering, a laser excitation with 532 nm was used, but this resulted in significant background fluorescence (in particular with particles from protein formulations, see below). Therefore, we proceeded with using 785 nm excitation where only minimal fluorescence was detected.

**Fig. 1:**
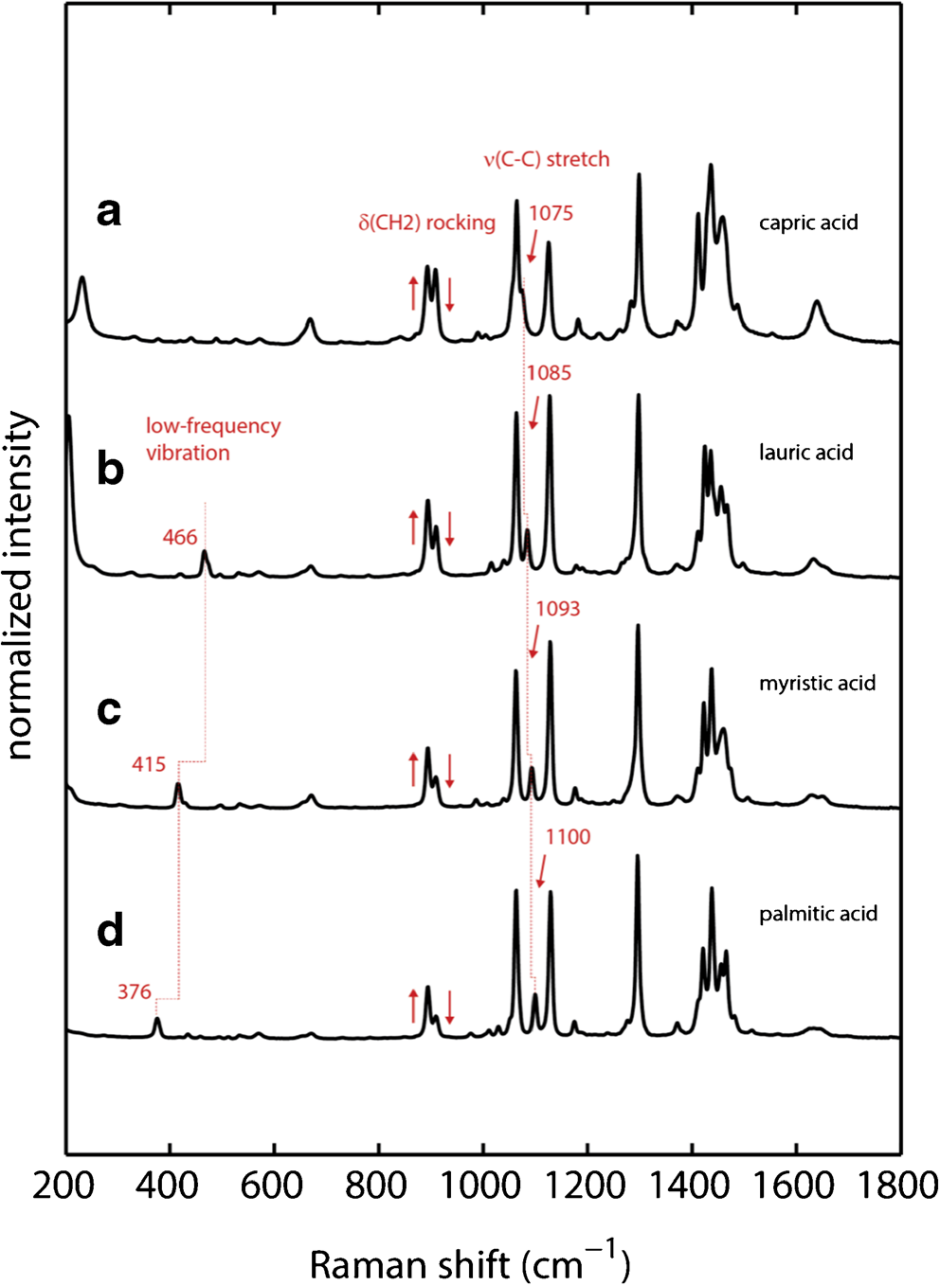
Experimental Raman spectra of dry fatty acids (*a*) capric acid (*b*) lauric acid (*c*) myristic acid and (*d*) palmitic acid. Experimental conditions: 4 mW laser power, T = 298 K, 60 s accumulation time.

Raman spectra of the dry fatty acids capric acid, lauric acid, myristic acid and palmitic acid were obtained using low laser power and are shown in Fig. 1. Characteristic for all fatty acids is the presence of two intense bands between 1050 and 1150 cm^−1^ arising from ν(C-C) stretch vibrations, one band at 1299 cm^−1^ arising from γ(CH2) twisting and a group of bands between 1400 and 1500 cm^−1^ caused by γ(CH2) wagging, γ(CH2) scissoring as well as δ(CH2) and δ(CH3) deformation vibrations (21). Typically carbonyl stretch frequencies of aliphatic compounds are found above 1700 cm^−1^. The carbonyl stretch frequency ν(C = O) of the fatty acids is red-shifted to 1640 cm^−1^ and shows only moderate Raman intensity indicating a local environment in which the carbonyl is hydrogen bonded (22). This peak is split into two peaks for lauric, myristic and palmitic acid suggesting heterogeneity of hydrogen bonding in the particle structure.

It can be seen that the spectra of the four fatty acids are highly similar. However, three characteristic marker bands can be identified helping to distinguish them. The first one is a ν(C-C) stretch vibration with moderate Raman intensity that is shifting consecutively from 1075 cm^−1^ in capric acid to 1085 cm^−1^ (lauric acid), 1093 cm^−1^ (myristic acid) and 1100 cm^−1^ in palmitic acid. The second notable difference is that the doublet originating from γ(CH2) rocking modes at 894 and 910 cm^−1^ is changing relative intensities with the band at 910 cm^−1^ decreasing in intensity when increasing the chain length of the fatty acid. The most prominent marker band is a low frequency vibration at 466 cm^−1^ in lauric acid which is red-shifted to 415 cm^−1^ in myristic acid and to 376 cm^−1^ in palmitic acid. This band is absent in capric acid. There are some minor differences in the spectral region between 1400 and 1500 cm^−1^ due to changes in the CH bending modes between the different fatty acids. However, this spectral region is very cluttered and does not allow for identification of fatty acids in complex mixtures. Therefore we only focus on the three marker bands described above.

### Effect of Water on the Raman Spectra

Our reference database contains spectra of dry fatty acids. Dry fatty acids typically exist in three major forms designated as α-, β- and γ-form, which mostly differ in the orientation of the side chains of the aligned fatty acids in their structure as well as slight changes in the hydrogen bonding of the acid headgroups or they can be amorphous (23,24). Most protein formulations are in aqueous solution and fatty acid particles most likely do not retain their initial dry state. In addition, particles resulting from mixtures of fatty acids might have partial disorder in their structure and different hydrogen bonding patterns. Therefore, we evaluated the effect of water on the Raman spectra. Hydrated fatty acid particles were filtered through a gold-coated polycarbonate filter and washed with water. Several effects in the spectra are observed including the disappearance of the band corresponding to the low frequency vibration between 376 and 466 cm^−1^ (Fig. S1). Another effect observed is that the relative intensities of the γ(CH2) rocking modes change with the band at 910 cm^−1^ increasing in intensity after the water wash. The last notable difference is a blue-shift of a γ(CH2) vibration at 1425 cm^−1^ resulting in an intense band around 1436 cm^−1^. It is worth noting that the characteristic marker band due to the ν(C-C) stretch vibration between 1075 and 1100 cm^−1^ does not change at all and can still be used to distinguish different amorphous fatty acids. The fact, that there are no changes in the carbonyl ν(C = O) stretch frequency, which is a highly localized mode, implies that the hydrogen bonding to the acid group in the particle structure is similar as in the reference compound. Since only some vibrations in the fingerprint region are affected by hydration this indicates that there are small structural changes of the fatty acid side chain orientations in the particle. Therefore, care must be taken when comparing experimental vibrational spectra with reference libraries because the microenvironment of the investigated molecule can change key features due to specific interactions or reorientation in the particle structure. Nevertheless, there are still features in the spectra that are not affected by hydration and can be used for identification of similar fatty acids such as the characteristic ν(C-C) stretch vibration between 1075 and 1100 cm^−1^.

### Raman Spectroscopy at Low Temperatures

Melting points of the fatty acids are between 63°C for palmitic acid and 32°C for capric acid (25). In mixtures of fatty acids, the eutectic point will be lower than the melting point of the component with the lowest melting point. We found that Raman experiments carried out at room temperature resulted in melting of the particles due to the heat created by the laser. Keeping the sample at low temperature increases stability of labile particles and allows for higher power laser illumination to obtain better signal to noise. Therefore, the possibility of low temperature Raman experiments to provide stabilizing conditions was evaluated. In addition, lower temperature usually results in narrower spectral linewidths and hence improved resolution. However, at low temperatures reorientation of molecules within the particle might occur, especially due to changes in hydrogen bond lengths and angles, causing bandshifts due to these specific changes. Control experiments were performed to evaluate if the identified marker bands are affected by temperature changes. The focus will be on the ν(C-C) stretch vibration between 1075 and 1100 cm^−1^ because this marker band was not affected by changes in hydration and/or crystal structure in contrast to the γ(CH2) rocking and low frequency vibrations between 376 and 466 cm^−1^ (Fig. S1). No significant bandshifts were observed when changing the temperature over the entire range from −40°C to −120°C (<1 cm^−1^ total change).

As mentioned previously, decreased linewidth allows better resolution to more readily assess mixtures of highly similar fatty acids. The linewidth of the ν(C-C) stretch marker band decreases from 10 cm^−1^ at 20°C to 8 cm^−1^ at −40°C for all fatty acids (Fig. S3). No further improvement is achieved by decreasing the temperature down to −120°C. For this reason, all low temperature experiments were performed at −40°C.

### Calculated Raman Spectra of Fatty Acids

To further distinguish similar polysorbate 20 degradation products, density functional theory (DFT) was used to calculate the vibrational spectra associated with various fatty acids that could potentially arise from polysorbate 20 degradation. Previous DFT studies have demonstrated that reasonable agreement between *in vacuo* calculated and experimentally observed vibrational frequencies can be obtained using harmonic approximation for a wide range of compounds (26,27). In this case, vibrational frequencies are usually overestimated and have to be scaled using empirical factors for certain combinations of functional and basis set. We have chosen a wavelength linear scaling procedure described by Yoshida *et al.* for correction of anharmonicity, which has been previously used for fatty acids (19,23).

Non-linear molecules have 3N-6 vibrational degrees of freedom, where N depicts the number of atoms. Capric acid has 32 atoms resulting in 90 normal modes and palmitic acid 50 atoms resulting in 144 normal modes. Hence, the vibrational spectra are complex. A comprehensive normal mode analysis for γ–form of oleic acid using DFT has been done in previous work and our analysis is based on these results (23).

**Fig. 2:**
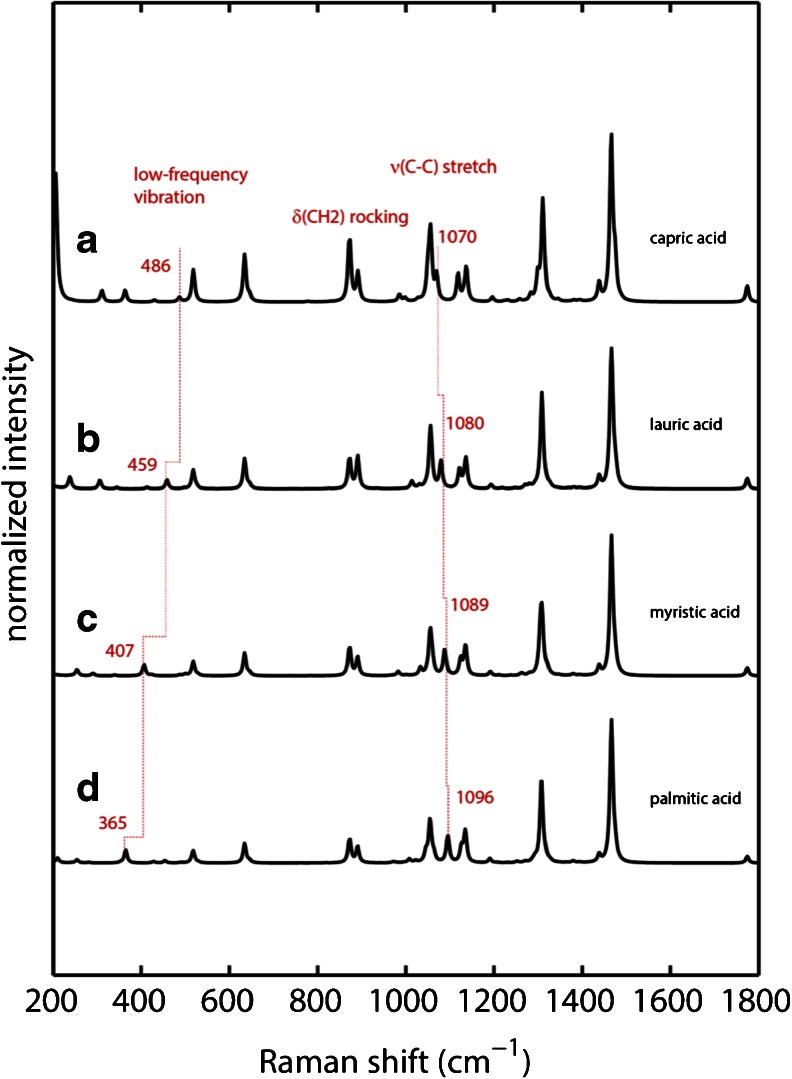
Calculated Raman spectra of fatty acids using DFT. Spectra have been scaled using a wavenumber linear scaling procedure according to Yoshida *et al.* (19) (*a*) capric acid (*b*) lauric acid (*c*) myristic acid and (*d*) palmitic acid.

The calculated Raman spectra are shown in Fig. 2. In general the calculations are able to reproduce experimental vibrational frequencies and intensities reasonably well when compared to experimental spectra shown in Fig. 1 especially in the spectral region above 800 cm^−1^. Vibrational frequencies for δ(CH2) rocking modes, ν(C-C) stretch modes and δ(CH2) twisting mode are all within ±20 cm^−1^ compared to experimental values (see Table I). The biggest discrepancy can be seen for the carbonyl stretch ν(C = O), which is blue-shifted to 1774 cm^−1^ in the calculated spectra. The fact that these samples consist of solid-state particles on filters contributes significantly to the sharpness of the experimental spectra, in which band broadening due to solution dynamics is not observed. Further, band shifts are negligible due to the hydrophobic nature of fatty acids which create negligible electric field contributions. The discrepancy in the carbonyl stretch frequency is attributed to the fact that specific interactions such as hydrogen bonding are not included in the gas-phase calculations.

**Table I:** Summary of Experimental and Calculated Vibrational Frequencies for the Specific Marker Bands and the Strong Bands in the Raman Spectra

	Capric acid		Lauric acid		Myristic acid		Palmitic acid	
	Exp.	Calc.	Exp.	Calc.	Exp.	Calc.	Exp.	Calc.
Low-frequency vibration	488	486	466	459	415	407	376	365
δ(CH2) rocking	893	872	893	872	893	872	893	872
δ(CH2) rocking	908	891	908	891	908	891	908	891
ν(C-C) stretch	1065	1057	1064	1057	1064	1057	1064	1057
ν(C-C) stretch	1075	1070	1085	1080	1093	1089	1100	1096
ν(C-C) stretch	1126	1137	1127	1137	1129	1137	1129	1137
δ(CH2) twisting	1299	1311	1298	1309	1297	1308	1296	1308
ν(C=O) stretch^a^	1640	1774	1633 (1657)	1774	1630 (1649)	1774	1630 (1646)	1774

^a^The large discrepancy is due to the fact that the carbonyl group is not hydrogen bonded in the gas-phase calculations

Comparison of the calculated spectra of all four fatty acids shows that they are very similar (as are the experimental spectra). The calculations are able to predict the presence of the same unique marker bands that were found in the experimental spectra. The ν(C-C) stretch mode that is shifting upon chain extension from 1075 cm^−1^ in capric acid to 1100 cm^−1^ in palmitic acid is predicted to be shifting from 1070 to 1096 cm^−1^. The same is true for the low-frequency vibration at 466 cm^−1^ in lauric acid that is red-shifted to 376 cm^−1^ in palmitic acid where calculations show this band red-shifting from 459 to 365 cm^−1^. Calculations indicate the presence of this band in capric acid to be at 486 cm^−1^ with low Raman intensity. This might explain the lack of this band in the experimental spectrum of capric acid because its predicted Raman activity is very low.

In conclusion, DFT calculations are able to reproduce vibrational frequencies reasonably well. However, the ability of the calculations to reproduce the Raman intensities at each frequency was challenging as seen for the δ(CH2) rocking modes. Raman intensities are calculated from the derivative of the molecular polarizability, and it is known that reliable intensities depend mostly on the size and quality of the basis set (20). The use of a large basis set enabled relatively accurate reproduction of the overall relative intensities in the spectra, but not the subtle changes in intensities seen for the δ(CH2) rocking modes. In general, DFT calculations can be used to predict the presence of specific marker bands and can aid in their assignment in experimental spectra. They are particularly useful to distinguish highly similar molecules such as fatty acids or fatty acid esters and can support mechanistic interpretations.

### Raman Spectra of Common Particles Found in Protein Formulations

**Fig. 3:**
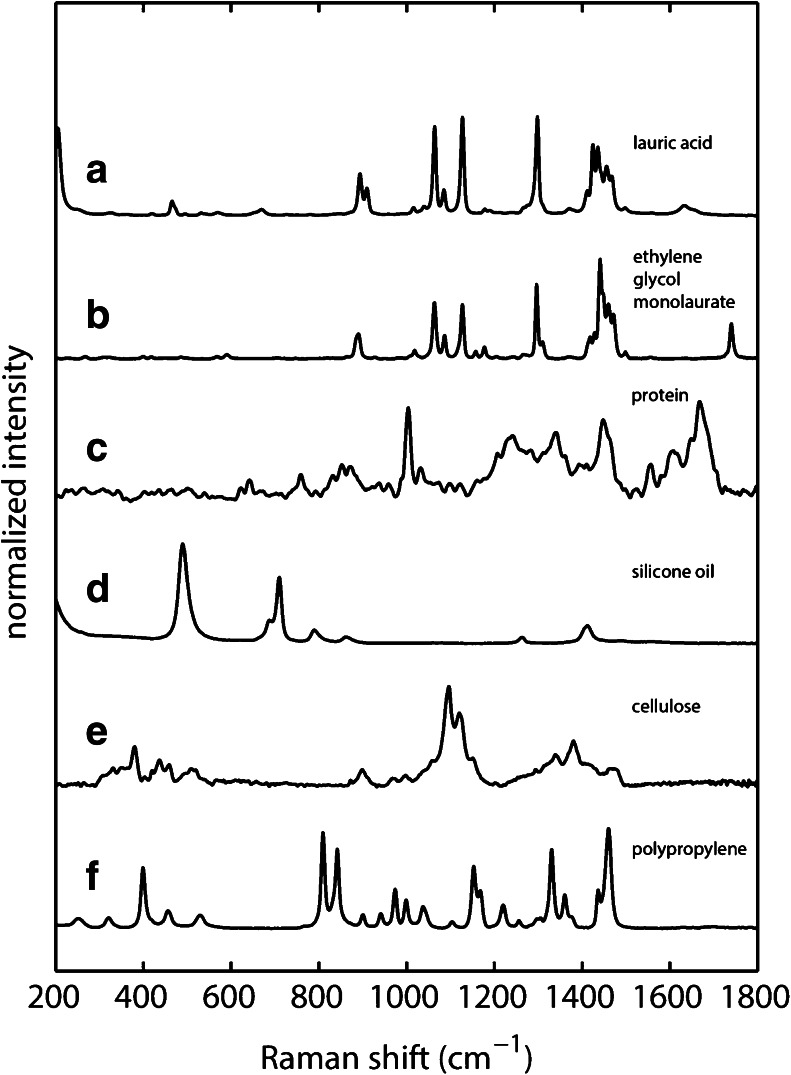
Raman spectra of common particulates and compounds found in biopharmaceutical formulations (*a*) lauric acid (*b*) ethylene glycol monolaurate (*c*) mechanically stressed antibody (*d*) silicone oil (*e*) cellulose and (*f*) polypropylene. Experimental conditions: 4 mW laser power, T = 298 K, 60 s accumulation time.

This section describes the general advantage of Raman spectroscopy to distinguish between intrinsic, extrinsic as well as inherent particles. Most particles have distinct spectral features allowing for unambiguous identification (see below). Figure 3 shows Raman spectra of particles, which are likely to be found in antibody formulations. For comparison, trace (a) contains the spectrum of dry lauric acid, which is a degradation product of polysorbate 20 resulting from hydrolysis of the side chain. The spectral features have already been discussed in the previous section. Trace (b) shows the Raman spectrum of ethylene glycol monolaurate, which is one of the expected degradation products of polysorbate 20 caused by oxidation. Qualitatively, this spectrum is very similar to lauric acid. However, subtle differences make it possible to differentiate between both of them. The ν(C = O) stretch vibration is blue-shifted to 1740 cm^−1^ and has a small linewidth of 10 cm^−1^ indicating that the carbonyl group is not hydrogen bonding in the crystal structure in ethylene glycol monolaurate. The δ(CH2) rocking vibrations at 893 and 908 cm^−1^ are merged together into one broadened band at 890 cm^−1^. The most prominent differences are changes in these δ(CH2) rocking modes, which are red-shifted to 890 and 885 cm^−1^, and the absence of the characteristic low-frequency vibration in ethylene glycol monolaurate (466 cm^−1^ in lauric acid). Another unique feature of ethylene glycol monolaurate is the presence of a medium intensity band at 1158 cm^−1^, which can be attributed to a ν(C-C) stretch vibration. For comparison, the Raman spectrum of ethylene glycol monolaurate was calculated using DFT as well (Fig. S2). The most prominent differences to lauric acid are captured by the calculations as well, *i.e.,* changes in the rocking modes as well as the disappearance of the low-frequency vibration. There is very good agreement between the experimental and calculated carbonyl stretch vibration ν(C = O) further corroborating the experimental finding that the carbonyl group of ethylene glycol monolaurate is not hydrogen bonded in the crystal structure.

Under various stress conditions, protein aggregates often times can be found in formulations. Trace (c) shows the spectrum of a protein particle generated by stressing a frozen formulation containing an IgG1 therapeutic antibody using mechanical stress and thawing at room temperature. Even though antibodies have a lot of vibrational degrees of freedom, they have unique marker bands mostly arising from backbone and aromatic amino acid vibrations thus enabling unambiguous identification using Raman spectroscopy (28). All antibodies contain phenylalanine residues in the Fab and Fc fragments. Phenylalanine has a sharp band around 1000 cm^−1^ originating from a breathing mode of its aromatic ring. This mode is independent of its microenvironment and can be used for normalization of spectra of the same antibody (28). Around 1650 cm^−1^ a broad band arising mainly from ν(C = O) backbone vibrations can be found (amide I) (28). Another characteristic backbone mode arises from out-of-phase combination of δ(N-H) bending and ν(C-N) stretching vibrations around 1550 cm^−1^ (amide II).

A very common compound found in protein formulations is silicone oil used for lubrication of stoppers and injection devices such as syringes, cartridges and plungers. The Raman spectrum of silicone oil shows several strong low-frequency modes at 490 cm^−1^ and a doublet at 689 cm^−1^/710 cm^−1^. The band at 490 cm^−1^ arises from ν(Si-O-Si) stretch vibrations and the doublet at 689 cm^−1^/710 cm^−1^ from symmetric δ(Si-CH3) rocking vibrations (29).

Foreign particles can be introduced during manufacturing of the drug or insufficient sterility during sample handling. Particles in these cases often times are fibrous (cellulose-based), polymers or glass particles resulting from delamination. The Raman spectrum of cellulose is shown in Fig. 3 (trace e). One characteristic is the presence of a doublet at 1096 and 1123 cm^−1^ arising from a combination of ν(C-C) and ν(C-O) stretch vibrations (21). Most features in the spectrum are broadened, in particular bands between 1200 and 1400 cm^−1^ arising from δ(HCC), δ(HCO) and δ(HOC) bending vibrations. Polymer compounds are easily identified because their vibrational spectra are composed of highly resolved bands due to their higher order structure and organized environment. As an example we show the Raman spectrum of polypropylene which can be introduced into the sample *e.g.,* by storage containers or pipette tips. The strong transitions at 809 and 842 cm^−1^ can be assigned to δ(CH2) rocking vibrations (30). Other strong transitions arise at 1153 cm^−1^ (combination of ν(C-C) stretch and δ(CH2) bending), 1168 cm^−1^ (ν(C-C) stretch, δ(CH2) rocking and δ(CH2) wagging), and 1460 cm^−1^ (δ(CH2) bending) (30).

Since molecular identity is associated with a characteristic vibrational fingerprint, it can be seen that both highly diverse and very similar molecules can be distinguished using Raman spectroscopy. Most compounds can be identified unless they are metal based and have only low-frequency vibrations below the laser filter cutoff. In the next sections we focus on identification of insoluble polysorbate 20 degradation products because this is particularly challenging due to the properties of the particles, which are labile, temperature sensitive as well as heterogeneous.

### Identification of Degradation Products in Model Systems

To study the feasibility of Raman spectroscopy to identify fatty acid particles derived from polysorbate in complex mixtures, we digested 0.2 mg/mL polysorbate 20 with 30 ppm rabbit liver esterase at 40°C for 12 h. Esterases hydrolyse esters into fatty acids and alcohols. Polysorbate 20 contains a complex mixture of fatty acid side-chains with the predominant species lauric acid and myristic acid in a ~3.5:1 ratio (12). Therefore, most of the particles generated from enzymatic treatment of polysorbate 20 are expected to be composed of mixtures of these fatty acids, in particular of lauric acid and myristic acid.

**Fig. 4:**
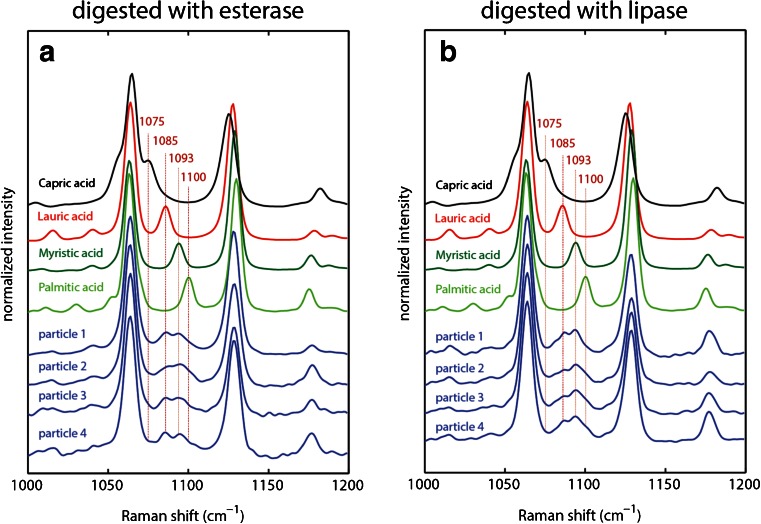
Experimental Raman spectra of fatty acid particles in model systems of polysorbate 20 digested with (**a**) rabbit liver esterase and (**b**) pancreatic lipase. Experimental conditions: T = 233 K, 16 mW laser power, 300 s accumulation time. For comparison the reference spectra of capric, lauric, myristic and palmitic acid are shown.

The spectra for several particles highlighting the range between 1000 and 1200 cm^−1^ are shown in Fig. 4A. The spectra showing the entire spectral range can be found in Fig. S6. It can be seen that the two strong bands arising from ν(C-C) stretches at 1065 and 1126 cm^−1^ are the same in all particles and do not allow for discrimination of different fatty acids. However, the marker band of the unique ν(C-C) stretch located between those two bands shows heterogeneity with main peaks at both 1085 and 1093 cm^−1^ in agreement with the unique ν(C-C) stretch vibrations found in lauric and myristic acid. No band at 1175 cm^−1^ is present indicating either the absence of capric acid or the possibility that most of the capric acid is still solubilized and not a constituent of the particles. Some particles show a small shoulder around 1100 cm^−1^ suggesting the presence of small amounts of palmitic acid. The ratio between lauric and myristic acid is ~1:1. Based on the relative abundance of lauric and myristic acid in polysorbate 20 as describend above (3.5:1 ratio between lauric and myristic acid) this can be interpreted in terms of their different solubility (short chain fatty acids have a higher solubility). So even though there will be more lauric acid side chains in the polysorbate 20 raw material it is likely that a larger fraction of it is solubilized and that the actual insoluble particles have larger contributions of the longer chain fatty acids. In conclusion, most insoluble particles on the filter are mixtures of lauric acid, myristic acid, and possibly small amounts of palmitic acid.

In a second model system, 0.2 mg/mL polysorbate 20 was digested with 30 ppm pancreatic lipase at 40°C for 12 h. Lipases convert triglyceride substrates to monoglycerides and fatty acids (31). When drying particles in a desiccator over phosphorous pentoxide in a manner similar to the previous experiment, it was discovered that most of the particles disappeared during the drying process. So far there is no obvious explanation for this but it might be due to the fact that the melting point of these particles was much lower than in the previous case. Therefore, we filtered particles and transferred the filter directly to the cold cryo-cell without any drying. The Raman spectra are shown in Fig. 4B and it can be seen that they are very similar to the spectra in the other model system. Most particles mainly consist of lauric acid and myristic acid with small contributions of palmitic acid.

To summarize, chemical identities of similar components in heterogeneous particles can be captured using Raman spectroscopy. If signal-to-nose is good enough it is even possible to estimate relative contributions of each fatty acid to the individual particles as stated above (~1:1 ratio between lauric and myristic acid).

### Identification of Degradation Products in Aged Antibody Formulations

After evaluating known model systems, we tested our methodology on aged antibody formulations containing particles of unknown identity. Two formulations were chosen that were aged at 5°C for 4 years (Mab1) and at 5°C for 6 years (Mab2). Polysorbate 20 quantification using HPLC assay with ELSD detection showed that 70% of all polysorbate 20 in Mab1 formulation and 42% in Mab2 formulation were intact (see Fig. S4). Although, protein particles were observed in these formulations (Fig. S5), we focus this discussion on polysorbate–related degradation products as the protein particles are readily distinguishable from fatty acids and fatty acid esters based on the features described in a previous section, *i.e.,* the amide bands and a strong phenylalanine band around 1000 cm^−1^.

In the first experiment, a filter containing particles from Mab1 formulation was transferred directly to the cold cryo-cell and spectra were acquired of two particles (particle 1 and particle 2). In a second experiment, the filter was dried over phosphorous pentoxide for 2 h before transferring it to the cryo-cell, and data was acquired (particles 3–5) for comparison against the non-dried sample. Similar to the results obtained from the model systems, it can be seen that the that the two strong bands arising from ν(C-C) stretches at 1065 and 1126 cm^−1^ are similar in each particle and do not allow for discrimination (full range spectra are shown in Fig. S7). The marker band in between these strong bands shows heterogeneity as well. However, when compared with the model systems, the particles in the Mab formulation are primarily composed of the longer chain fatty acids palmitic and myristic acid rather than lauric and myristic acids as observed in the model systems. The characteristic band at 1158 cm^−1^ arising from the unique ν(C-C) stretch vibration in ethylene glycol monolaurate is missing. The presence of the characteristic doublet of the δ(CH2) rocking modes in the full range spectra further confirms that the particles are fatty acids rather than fatty acid esters (Fig. S7). In conclusion, there was no difference in particle identity between dried sample and sample containing residual water indicating that the drying process did not change particle composition.

**Fig. 5:**
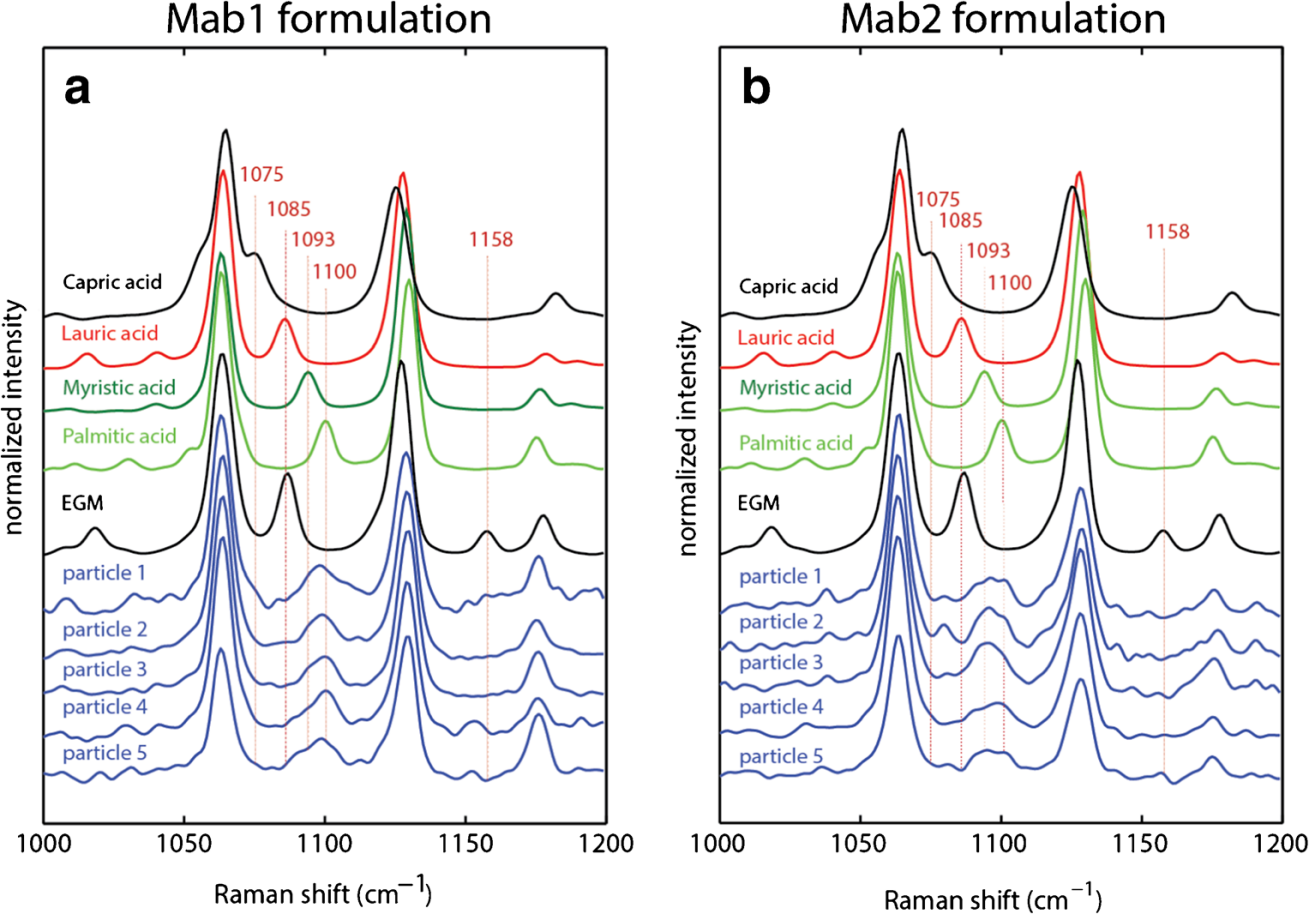
Experimental Raman spectra of isolated particles from Mab1 and Mab2 formulations. For comparison the reference spectra of capric, lauric, myristic, palmitic acid and ethylene glycol monolaurate (EGM) are shown. Experimental conditions: (**a**) T = 233 K, 16 mW laser power, 300 s accumulation time. Particle 1 and 2 were transferred directly to the cold Raman cell. Particles 3–5 were dried over phosphorous pentoxide for 2 h before measurement (**b**) T = −40°C, 4 mW laser power, 90 s accumulation time. Particles were dried over phosphorous pentoxide for 2 h before measurement.

Particles from Mab2 formulation were dried over phosphorous pentoxide for 2 h before measurement. The spectra are similar to the ones obtained from particles in Mab1 formulation (Fig. 5b). The chemical identity of the particles is comprised mainly of palmitic and myristic acid. In a control experiment, particles from Mab2 formulation were dried overnight over phosphorus pentoxide. After drying, partial recovery of the low-frequency vibration at 376 cm^−1^ was observed consistent with the presence of palmitic acid, which showed similar behavior (Fig. S8). No further recovery was achieved by longer drying times. Unfortunately, drying under these conditions leads to loss of short chain fatty acids due to their higher volatility. Therefore, we do not recommend prolonged drying of particles. To recover spectra identical to the library spectra shown in Fig. 1, it may be necessary to perform a temperature jump and recrystallize the fatty acids back to their original crystal structure.

In general, these experiments demonstrate that identification of different components in mixtures of particles in the size range 10–80 μm is possible based on the presence of unique spectral features. Since molecules in particles may be amorphous and experience a different microenvironment than in its original dry structure, careful control experiments coupled with DFT calculations should be included to assure that these changes in vibrational spectra are captured by the operator.

### Implications for the Mechanism of Degradation

As mentioned previously, the mechanism of polysorbate degradation in protein formulations is still under debate. It is believed that hydrolysis is one of the major mechanisms at low temperature while oxidation is accelerated by stress conditions such as elevated temperature or prolonged light exposure, but there is no conclusive evidence yet (12). Since both pathways create different degradation products the chemical nature of the insoluble particles can help reveal the mechanism of degradation.

We investigated insoluble particles in two different Mab formulations stored at low temperature for extended period. Raman spectroscopy confirmed that the particles consist of fatty acids rather than fatty acid esters. In addition, particles are comprised of mixtures of the long chain fatty acids palmitic and myristic acid and not of the short chain fatty acids lauric and capric acid. This can be explained based on the much lower solubility of the longer chain fatty acids, which preferentially precipitate out in solution while the short chain fatty acids might be still solubilized. There was no evidence for the presence of insoluble particles comprised of fatty acid esters (note that fatty acid esters can still be present in solubilized form). Therefore, our findings indicate that hydrolysis is the predominant mechanism leading to particle formation under these experimental conditions.

## Conclusion

Surfactant degradation in biopharmaceutical formulations is one of the processes that can contribute to insoluble particulate formation because hydrophobic degradation products of surfactant usually have low solubility in formulation buffer. Polysorbate 20 can degrade *via* hydrolysis or oxidative damage resulting in different degradation products and particulate matter. Information about the chemical identity of the resulting particles enables elucidation of the major degradation pathway leading to particle formation and may aid in prevention of these processes.

In the present study, we exploited the potential of dispersive Raman spectroscopy for identification of degradation products of polysorbate 20. Raman spectroscopy is well suited for studying biological systems because water shows only very weak Raman activity. Its biggest drawbacks include background fluorescence due to impurities in the formulations, which can be minimized by using 785 nm excitation, as well as the possibility of laser-induced photodamage of the sample.

In general, insoluble particles resulting form polysorbate 20 degradation are fatty acids or fatty acid esters, all of which are highly similar molecules. However, since each molecule is slightly different, it exhibits unique vibrational fingerprints allowing for discrimination. We performed Raman spectroscopy on purified fatty acids to identify these unique vibrations and evaluated different hydration states to ensure positive identity. Analysis was made quantitative by comparing experimental spectra with calculated spectra using DFT in which it was possible to identify the unique vibrations as well. Model systems with known composition of degradation products demonstrated that it is possible to disentangle mixtures of fatty acids using Raman spectroscopy. Finally, fatty acids were identified as the main type of particles in two different antibody formulations, which were aged at 5°C for several years. These particles were mainly composed of the long chain fatty acids myristic and palmitic acid, whereas the particles in the model systems were mainly composed of lauric and capric acid. This indicates that under these conditions, polysorbate hydrolysis is the predominant mechanism for particle formation.

In conclusion, Raman spectroscopy is a powerful tool to identify particles in protein formulations. Its advantage compared to FTIR spectroscopy is based on the fact that there is no interference by water and use of high power lasers enables identification of much smaller particles (down to 0.5 μm depending on the optical properties of the particle). One drawback, however, is absolute quantification of each fatty acid component in the particles due to the very high similarity of the vibrational spectra and since the Raman process itself is not based on absorption of light but on scattering.

Further studies are ongoing and focus on different product-related particles to identify reasons for instability of excipients. Especially challenging is particle identification in fairly stable formulations because of the limited number and small size of particles present. In these cases, Raman spectroscopy is the method of choice for chemical identification due to its higher sensitivity compared to FTIR spectroscopy. Knowledge of degradation pathways enables the manufacturer to avoid them and improve overall product quality and safety.

## Electronic supplementary material

Below is the link to the electronic supplementary material.

ESM 1 Temperature dependence of Raman spectra. Calculated spectrum of ethylene glycol monolaurate. Effect of water on Raman spectra of fatty acids. Raman spectra of isolated fatty acid particles showing the entire spectral range. ELSD HPLC data of Mab formulations. (DOCX 2494 kb)

## Abbreviations

DFT: Density functional theory
ELSD: Evaporative light scattering detector
HPLC: High performance liquid chromatography
Mab: Monoclonal antibody

## References

[1] Demeule B, Messick S, Shire SJ, Liu J (2010). Characterization of particles in protein solutions: reaching the limits of current technologies. AAPS J.

[2] Carpenter JF, Randolph TW, Jiskoot W, Crommelin DJ, Middaugh CR, Winter G (2009). Overlooking subvisible particles in therapeutic protein products: gaps that may compromise product quality. J Pharm Sci.

[3] Rosenberg A (2006). Effects of protein aggregates: an immunologic perspective. AAPS J.

[4] Singh SK, Afonina N, Awwad M, Bechtold-Peters K, Blue JT, Chou D (2010). An industry perspective on the monitoring of subvisible particles as a quality attribute for protein therapeutics. J Pharm Sci.

[5] Cromwell MEM, Hilario E, Jacobson F (2006). Protein aggregation and bioprocessing. AAPS J.

[6] Patel AR, Lau D, Liu J (2012). Quantification and characterization of micrometer and submicrometer subvisible particles in protein therapeutics by use of a suspended microchannel resonator. Anal Chem.

[7] Filipe V, Hawe A, Jiskoot W (2010). Critical evaluation of Nanoparticle Tracking Analysis (NTA) by nanosight for the measurement of nanoparticles and protein aggregates. Pharm Res-Dordr.

[8] Narhi LO, Jiang YJ, Cao S, Benedek K, Shnek D (2009). A critical review of analytical methods for subvisible and visible particles. Curr Pharm Biotechnol.

[9] Werk T, Volkin DB, Mahler HC (2014). Effect of solution properties on the counting and sizing of subvisible particle standards as measured by light obscuration and digital imaging methods. Eur J Pharm Sci Off J Eur Fed Pharm Sci.

[10] Zölls S, Tantipolphan R, Wiggenhorn M, Winter G, Jiskoot W, Friess W (2012). Particles in therapeutic protein formulations, Part 1: overview of analytical methods. J Pharm Sci.

[11] Wartewig S, Neubert RHH (2005). Pharmaceutical applications of Mid-IR and Raman spectroscopy. Adv Drug Deliv Rev.

[12] Kerwin BA (2008). Polysorbates 20 and 80 used in the formulation of protein biotherapeutics: Structure and degradation pathways. J Pharm Sci.

[13] Kishore RK, Kiese S, Fischer S, Pappenberger A, Grauschopf U, Mahler H-C (2011). The Degradation of Polysorbates 20 and 80 and its Potential Impact on the Stability of Biotherapeutics. Pharm Res-Dordr.

[14] Cao X, Fesinmeyer RM, Pierini CJ, Siska CC, Litowski JR, Brych S, et al. Free fatty acid particles in protein formulations, Part 1: microspectroscopic Identification. J Pharm Sci. 2014;104(2):433–46. 10.1002/jps.24126 25175016

[15] Hewitt D, Zhang T, Kao Y-H (2008). Quantitation of polysorbate 20 in protein solutions using mixed-mode chromatography and evaporative light scattering detection. J Chromatogr A.

[16] Frisch MJ, Trucks GW, Schlegel HB, Scuseria GE, Robb MA, Cheeseman JR (2009). Gaussian 09.

[17] Becke AD (1993). Density‐functional thermochemistry. III. The role of exact exchange. J Chem Phys.

[18] Lee C, Yang W, Parr RG (1988). Development of the Colle-Salvetti correlation-energy formula into a functional of the electron density. Phys Rev B.

[19] Yoshida H, Takeda K, Okamura J, Ehara A, Matsuura H (2002). A new approach to vibrational analysis of large molecules by density functional theory: wavenumber-linear scaling method†. J Phys Chem A.

[20] Kaminski S, Gaus M, Phatak P, von Stetten D, Elstner M, Mroginski MA (2010). Vibrational Raman spectra from the self-consistent charge density functional tight binding method via classical time-correlation functions. J Chem Theory Comput.

[21] De Gelder J, De Gussem K, Vandenabeele P, Moens L (2007). Reference database of Raman spectra of biological molecules. J Raman Spectrosc.

[22] Fried SD, Bagchi S, Boxer SG (2013). Measuring electrostatic fields in both hydrogen-bonding and non-hydrogen-bonding environments using carbonyl vibrational probes. J Am Chem Soc.

[23] Mishra S, Chaturvedi D, Kumar N, Tandon P, Siesler HW (2010). An ab initio and DFT study of structure and vibrational spectra of γ form of Oleic acid: comparison to experimental data. Chem Phys Lipids.

[24] Kaneko F, Yano J, Sato K (1998). Diversity in the fatty-acid conformation and chain packing of cis-unsaturated lipids. Curr Opin Struct Biol.

[25] Haynes WM (2013). CRC handbook of chemistry and physics.

[26] Rauhut G, Pulay P (1995). Transferable scaling factors for density functional derived vibrational force fields. J Phys Chem.

[27] Merrick JP, Moran D, Radom L (2007). An evaluation of harmonic vibrational frequency scale factors. J Phys Chem A.

[28] Barth A, Zscherp C (2002). What vibrations tell about proteins. Q Rev Biophys.

[29] Cai D, Neyer A, Kuckuk R, Heise HM (2010). Raman, mid-infrared, near-infrared and ultraviolet–visible spectroscopy of PDMS silicone rubber for characterization of polymer optical waveguide materials. J Mol Struct.

[30] Nielsen AS, Batchelder DN, Pyrz R (2002). Estimation of crystallinity of isotactic polypropylene using Raman spectroscopy. Polymer.

[31] Svendsen A (2000). Lipase protein engineering. Biochim Biophys Acta Protein Struct Mol Enzymol.

