# Supplementary material for: Identification of Subvisible Particles in Biopharmaceutical Formulations Using Raman Spectroscopy Provides Insight into Polysorbate 20 Degradation Pathway
Source: Pharm Res. 2015 Mar 14;32(9):2877–88. doi: 10.1007/s11095-015-1670-x (PMC4526581; doi:10.1007/s11095-015-1670-x)

**Identification of subvisible particles in biopharmaceutical formulations using Raman spectroscopy provides insight into polysorbate 20 degradation pathway**

Miguel Saggu*, Jun Liu and Ankit Patel

Late Stage Pharmaceutical Development, Genentech Inc., South San Francisco, CA 94080

* To whom correspondence should be addressed: saggu.miguel@gene.com

**Supporting Information**

**Figures**

**Figure S1**

Raman spectra of dry fatty acids and changes upon washing with water. (a) Capric acid (b) Lauric acid (c) Myristic acid (d) Palmitic acid (e) Ethylene glycol monolaurate. Experimental conditions: 4 mW laser power, T = 298 K, 60 s accumulation time.


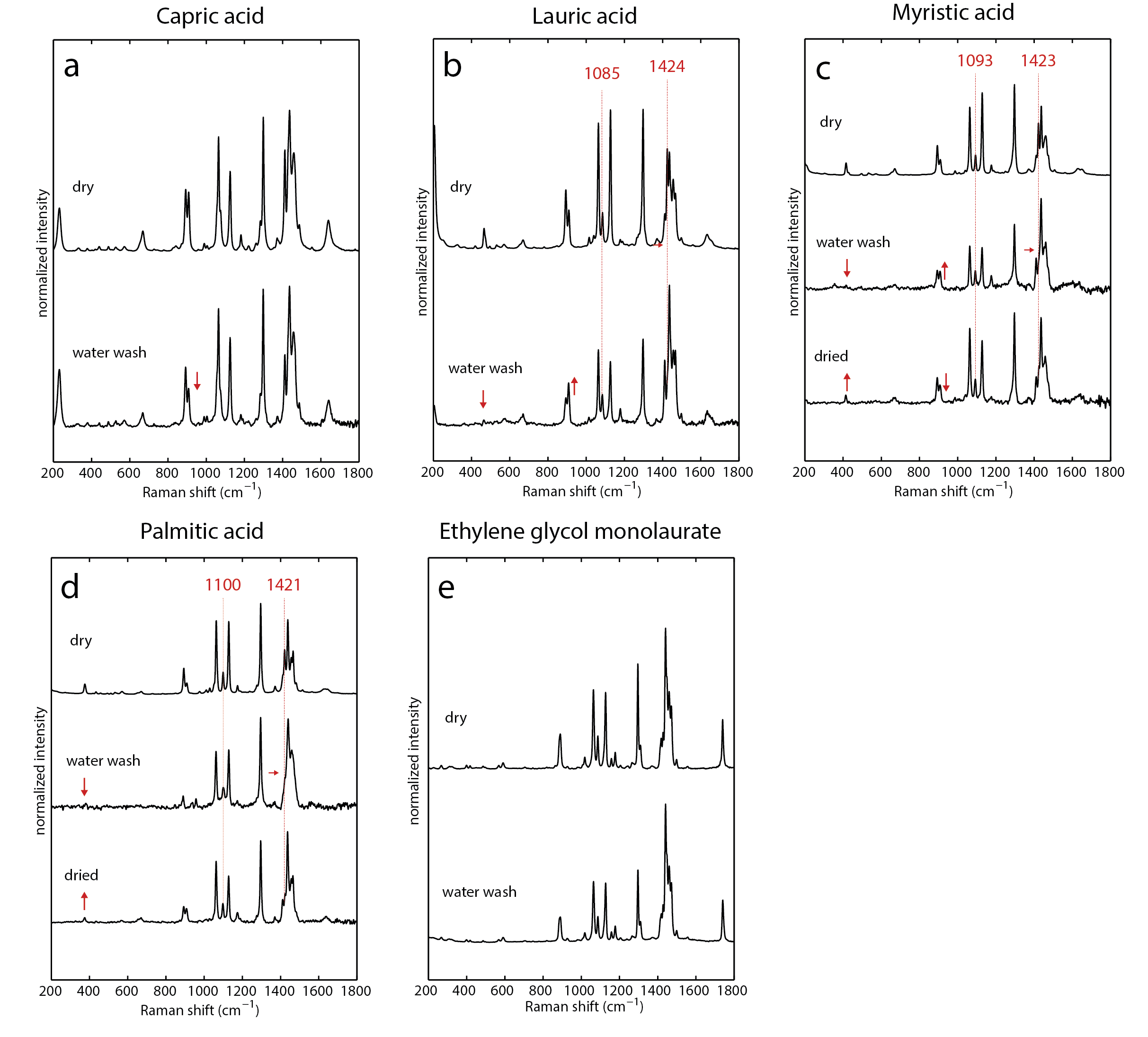


**Figure S2**

Experimental (red) and calculated (black) Raman spectra of (a) dry lauric acid and (b) dry ethylene glycol monolaurate.


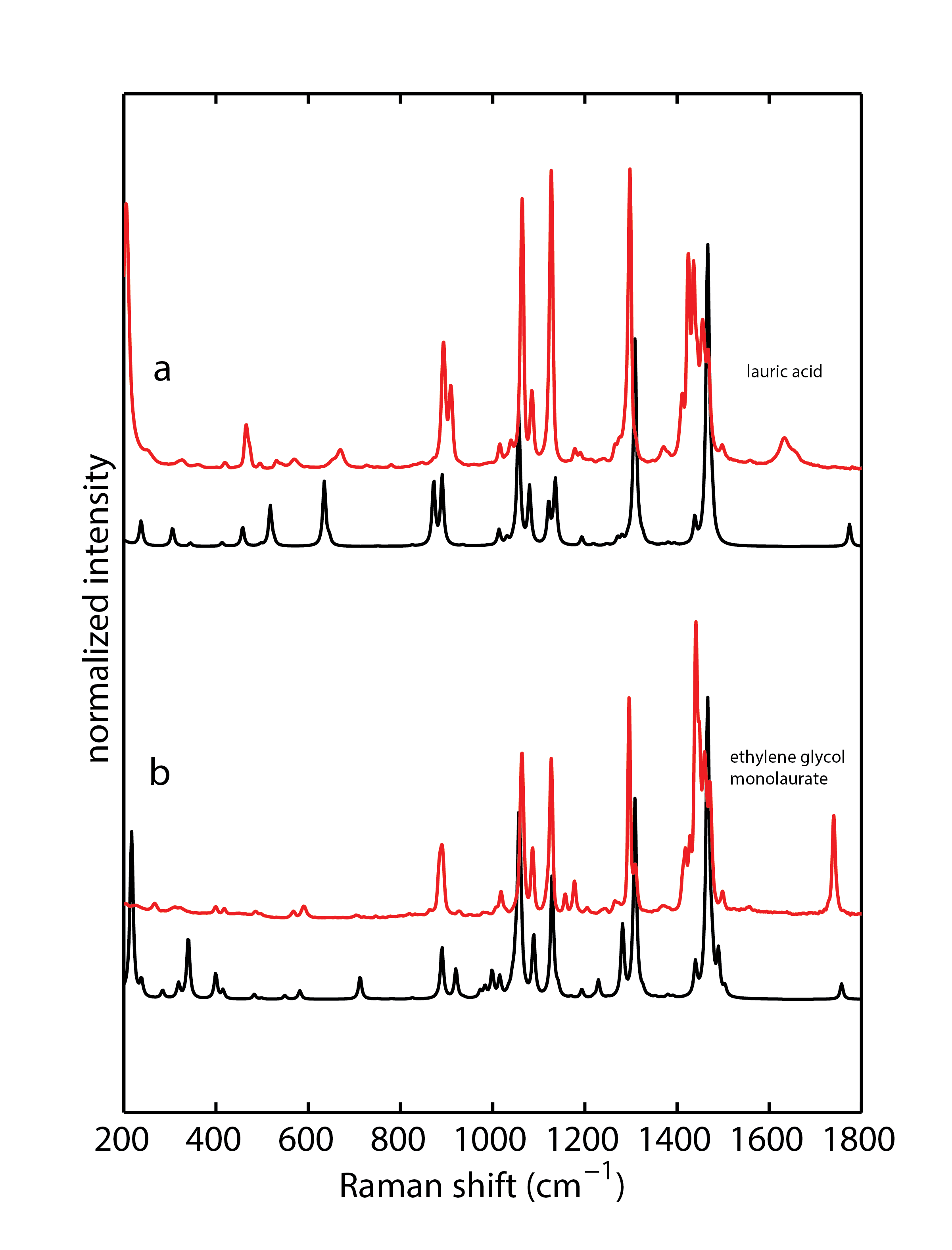


**Figure S3**

Effect of temperature on Raman spectra of dry fatty acids between 20°C and -120°C.

Experimental conditions: 4 mW laser power, 60 s accumulation time.


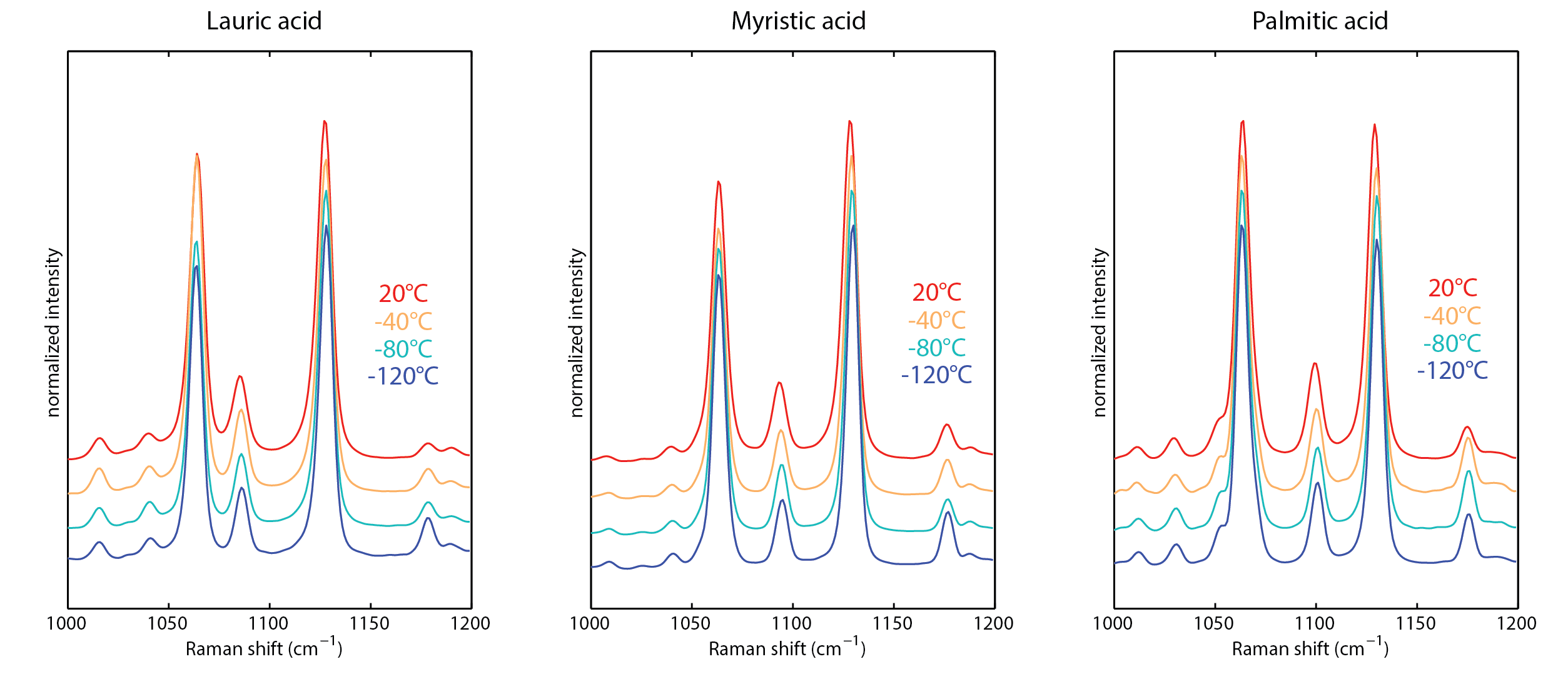


**Figure S4**

HPLC traces of Mab formulations for quantification of intact polysorbate 20. Each sample was run in duplicate. The black trace represents a standard containing 0.02% polysorbate 20, which corresponds to the initial amount of polysorbate 20 in both formulations.


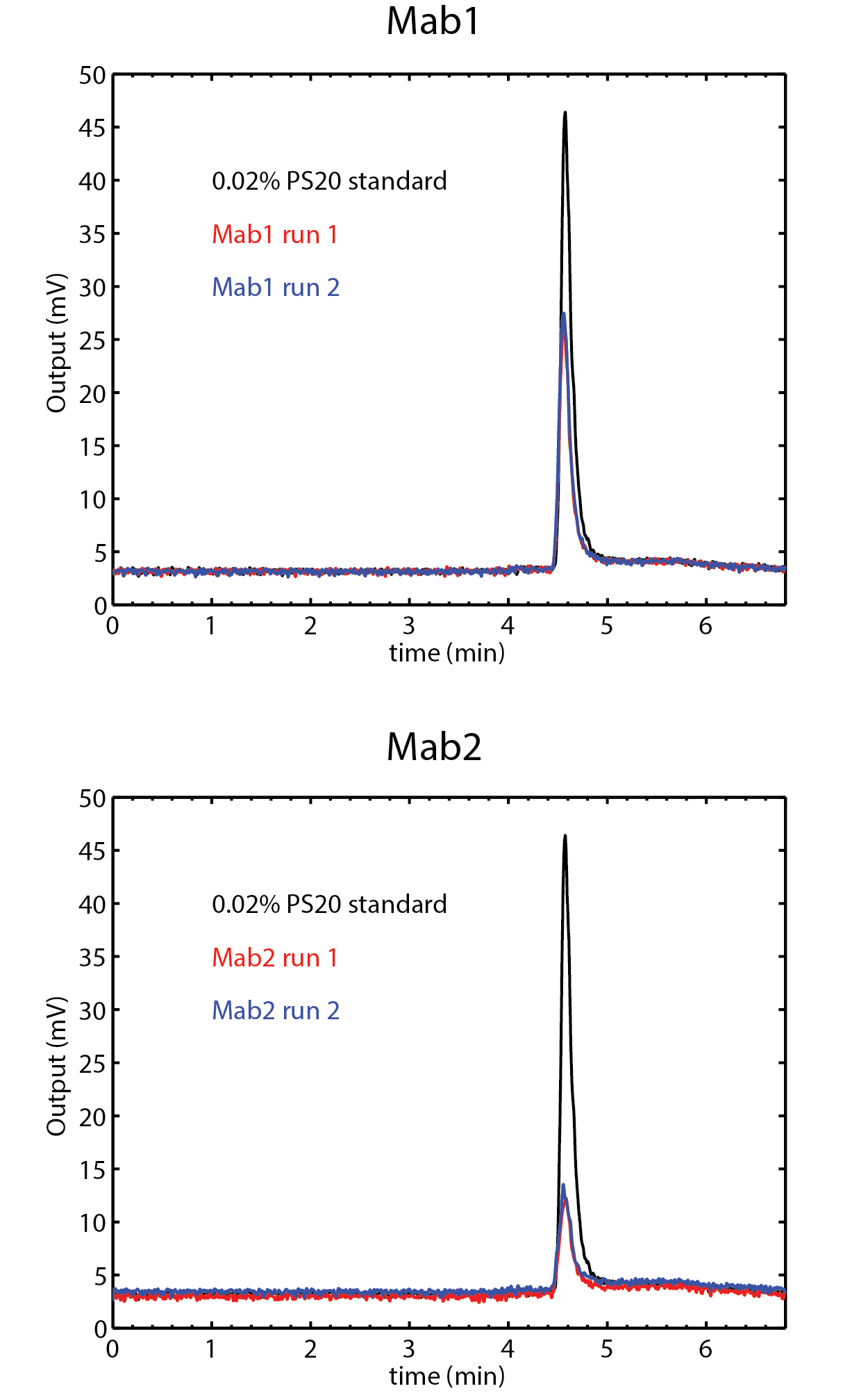


**Figure S5**

Proteinaceous particles found in Mab1 formulation and reference spectra. (a) Lauric acid reference (b) protein reference (c) particle 1 (d) particle (2). The regularly sized dark circles are 5 µm pores of the underlying filter. Spectra are from particles within the area of the red circles. Experimental conditions: T = 233 K, 16 mW laser power, 300 s accumulation time.

**
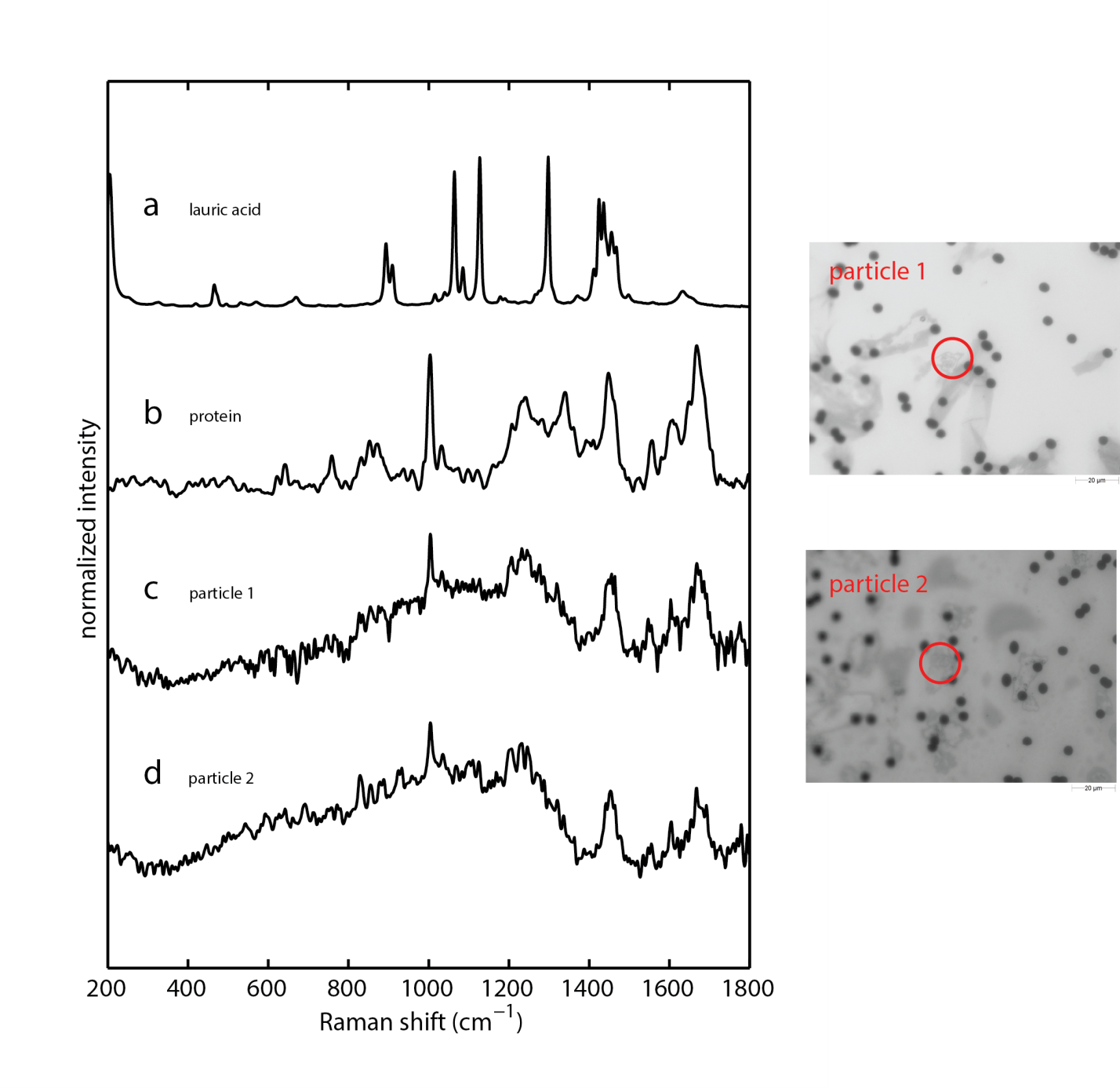
**

**Figure S6**

Images of the particles found in esterase digested polysorbate 20 and investigated with Raman spectroscopy. The regularly sized dark circles are 5 µm pores of the underlying filter. In addition the full range Raman spectra obtained from particles within the area of the red circles are shown. Experimental conditions: T = 233 K, 16 mW laser power, 300 s accumulation time.


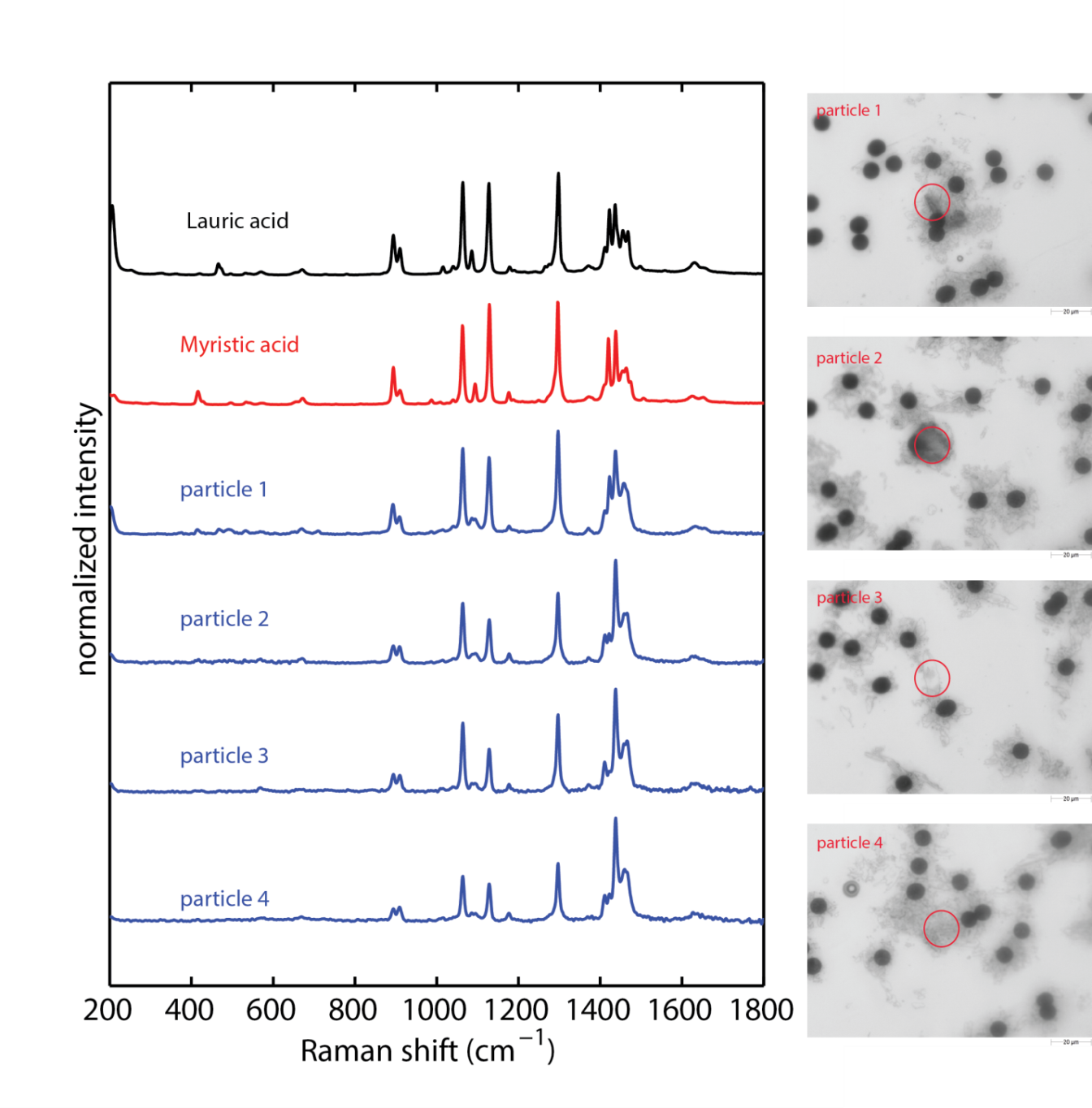


**Figure S7**

Images and full range Raman spectra of particles from Mab1 formulation. The regularly sized dark circles are 5 µm pores of the underlying filter. Full range Raman spectra obtained from particles within the area of the red circles are shown. Experimental conditions: T = 233 K, 16 mW laser power, 300 s accumulation time.

**
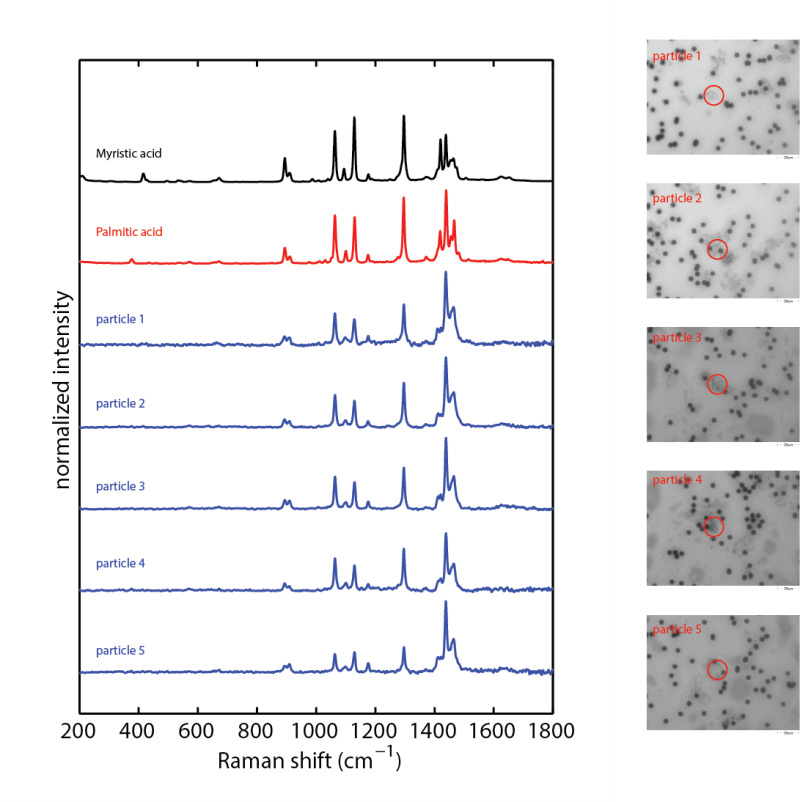
**

**Figure S8**

Particles of Mab2 formulation dried overnight over phosphorous pentoxide. Experimental conditions: T = -40°C, 4 mW laser power, 90 s accumulation time.


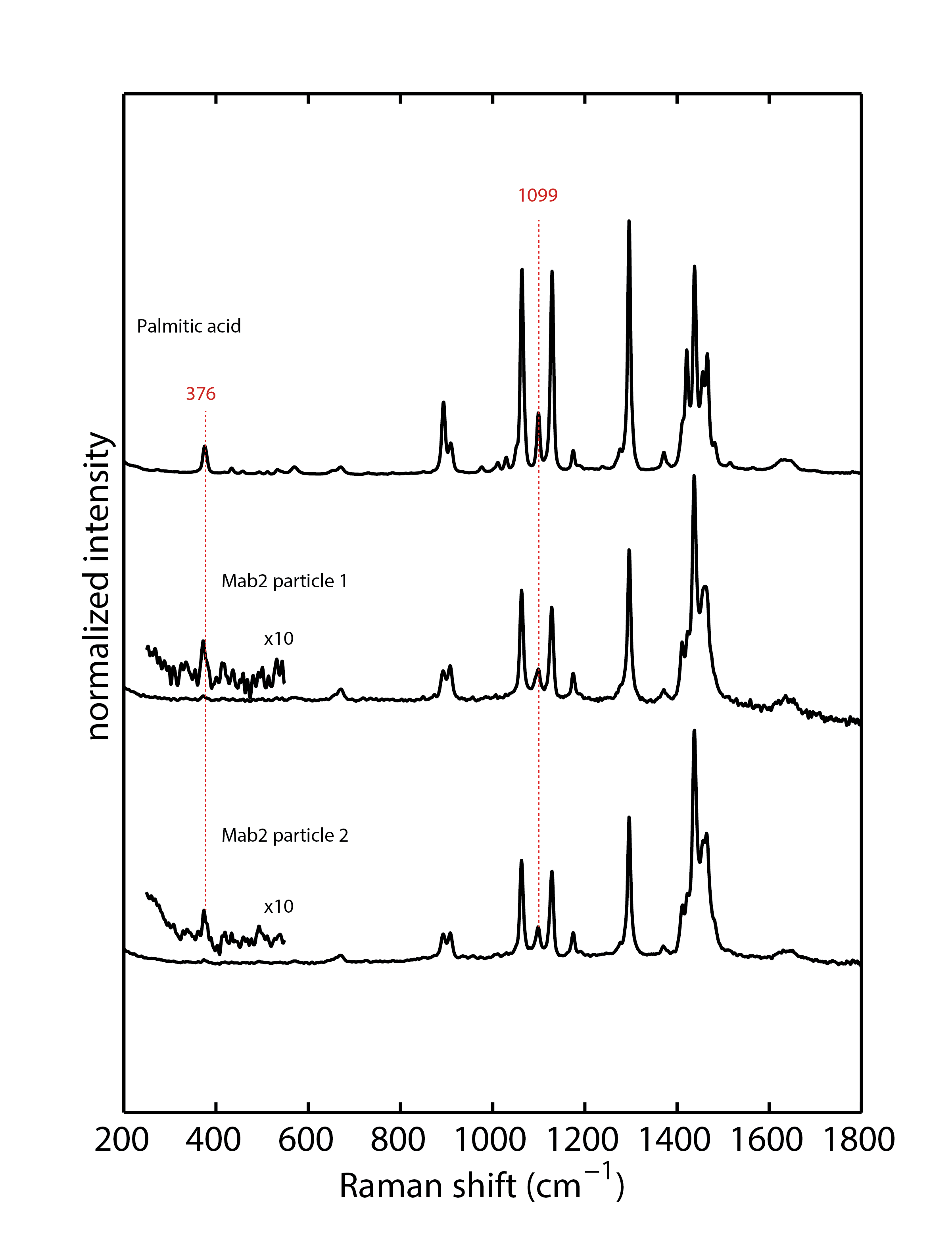

Supplement: Supplementary file 1 — Temperature dependence of Raman spectra. Calculated spectrum of ethylene glycol monolaurate. Effect of water on Raman spectra of fatty acids. Raman spectra of isolated fatty acid particles showing the entire spectral range. ELSD HPLC data of Mab formulations. (DOCX 2494 kb) [file 11095_2015_1670_MOESM1_ESM.docx]
